# The Abuse of Hospitals by Industrial Firms

**Published:** 1907-06-08

**Authors:** Fredk. W. Collingwood

**Affiliations:** Primrose Club, Park Place, W.


### THE ABUSE OF HOSPITALS BY INDUSTRIAL FIRMS.

Sir,—The modern practice of large industrial firms making a small annual payment to local hospitals, and in return for the same their well-paid employees receiving gratuitous surgical aid in case of trivial or semi-trivial accidents, is, I submit, a great mischief and grievance to practitioners who are willing to render good work for moderate fees. For if these well-paid employees are deemed fit objects of charity, the expense thereof should be borne by these wealthy firms, and members of the profession should not be exploited in this manner. Yours faithfully,

Fredk. W. Collingwood.

Primrose Club, Park Place, W., May 31.

